# Thromboembolism in nephrotic syndrome: controversies and uncertainties

**DOI:** 10.1016/j.rpth.2023.102162

**Published:** 2023-08-09

**Authors:** Kathrine Parker, Omar Ragy, Patrick Hamilton, Jecko Thachil, Durga Kanigicherla

**Affiliations:** 1 Manchester Institute of Nephrology and Transplantation, Manchester University NHS Foundation Trust, Manchester, United Kingdom; 2 Division of Pharmacy and Optometry, the University of Manchester, School of Health Sciences, Manchester Academic Health Science Centre, University of Manchester, Manchester, United Kingdom; 3 Wellcome Trust Centre for Cell Matrix Research, Division of Cell Matrix Biology and Regenerative Medicine, Manchester, United Kingdom; 4 Department of Haematology, Manchester University NHS Foundation Trust, Manchester, United Kingdom; 5 Division of Cardiovascular Sciences, the University of Manchester, School of Medical Sciences, Manchester, United Kingdom

**Keywords:** anticoagulants, arterial thromboembolism, glomerulonephritis, nephrotic syndrome, venous thromboembolism

## Abstract

Thromboembolism is one of the most serious complications of nephrotic syndrome, including both arterial and venous thromboembolic events. Rates of thromboembolism depend on a multitude of factors, including the severity and cause of nephrotic syndrome, with primary membranous nephropathy having the highest reported rates. In relation to arterial thromboembolism, the risk can be as high as 8 times that of an age- and sex-matched population. However, extrapolating risks is challenging, with published studies not being homogeneous, several being single center and retrospective, and including different causes of primary nephrotic syndrome.

Determining thromboembolic risk in nephrotic syndrome is essential to enable decision making on preventive strategies. However, lack of proven strategies to help estimate risk-benefit aspects underpins variations in clinical practice. Although the use of anticoagulation following a thrombotic event is clear, this still leaves us with a clinical dilemma as to if, and who, should receive prophylactic anticoagulation, with what agent, and for how long. In the absence of clear evidence to answer these questions, prophylactic anticoagulation strategies for nephrotic syndrome currently rely on expert consensus opinion, such as in the recently published 2021 Kidney Disease Improving Global Outcomes glomerular disease guidelines. In the mainstay, these recommendations relate to patients with membranous nephropathy.

Here, we detail the current controversies still faced by clinicians around the risk of thromboembolism in nephrotic syndrome, use of prophylactic anticoagulation in nephrotic syndrome and propose ways of advancing existing knowledge and practice in this field to unravel the conundrum.

## Essentials

• Arterial and venous thromboembolism are serious complications of nephrotic syndrome (NS).
• Multiple risk factors are associated with thromboembolism in NS.
• Uncertain who should receive thromboembolism prophylaxis, with what agent and for how long.
• We propose a decision scheme for anticoagulation in NS based on the current available evidence.

## Graphical abstract

**Figure:**
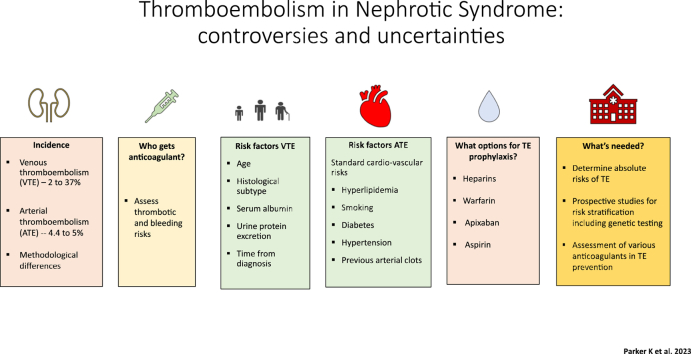

## How Much of a Problem is Thromboembolism in Nephrotic Syndrome?

Association of thromboembolism in proteinuric kidney disease was first described during the 19th century [1]. Venous thromboembolic (VTE) events encountered in nephrotic syndrome (NS) predominantly include thrombus burden in renal veins, deep veins of extremities, and pulmonary arteries. Description and rates of VTE vary widely between published studies, with incidence reported from 2% to 37%, Table [[2], [3], [4], [5], [6], [7], [8], [9], [10], [11], [12], [13], [14]]. Some of this variation can be attributed to study methodology, including inclusion criteria, duration of follow-up, and the use of screening investigations to identify thromboembolism [15].

**Table:** VTE events and risk factors for thromboembolism reported in studies of nephrotic syndrome over the last decade.

Author, country	Year	Context	Nephrotic syndrome	No.	Specific glomerulonephritis	TE events	Risk factors for TE
Li et al. [2] China^a^	2012	Prospective screening CTA	Yes	100	MN	Incidence, % VTE – 36 RVT – 33 (26 asymptomatic) PE – 17 (8 asymptomatic)	: Proteinuria • 7.98 g/d compared with 6.39 g/d had OR = 1.18 (95% CI, 1.02-1.35; *P* = .02) Ratio of proteinuria to serum albumin
Kumar et al. [3] UK^a^	2012	Retrospective Single Center	Most	101 78 primary	MN	Total numbers of events N: TE – 15 (19.2%) PE – 9 DVT – 3 RVT – 1 CVA – 1 Incidence VTE 7.6% (95% CI: 2.5-17.0) over 6 mo	Proteinuria o For every 1 g/d ↑ OR was 1.3 (95% CI: 1.05-1.58; *P* = .01) Serum Albumin o For every 1 g/dL ↑ OR was 0.8 (95% CI: 0.7-0.96; *P* = .01) Time from diagnosis
Barbour et al. [4] Canada^b^	2012	Prospective	20-70% IgAN 20% FSGS 50% MN 70%+	1313	Mixed MN 395 (30%) FSGS 370 (28%) IgAN 548 (42%)	Incidence MN – 7.9% FSGS – 3% IgAN – 0.4%	Histologic subtype NS o MN compared to IgAN RR 22 (95% CI5.3-92.1, *P* < .01) o FSGS vs IgAN; RR = 7.8 (95% CI: 1.7-35.2; *P* < .01) • After adjustment for baseline factors o MN vs IgAN 10.8 (95% CI 2.4-49.4; *P* = .002) o FSGS vs IgAN; RR = 59 Time from diagnosis • Days to 1st VTE was 151 in MN vs 453 in IgAN vs 1094 in FSGS Serum albumin <29 g/L; HR = 9.6; 95% CI:1.2-76.4; *P* = .03
Lionaki et al. [5] North America^a^	2012	Retrospective Registry based	Most	898	MN	VTE – 65 (7.2%) Incidence 0.017 per person-year (95% CI: 0.013, 0.021)	Serum albumin • 1.0 g/dL decrease in albumin associated with 2.13-fold increased risk of VTE (95% CI for adjusted OR: 1.32, 3.46; *P* = .002) • Albumin <2.0 g/dL compared with ≥3.0 g/dL OR = 3.56 (95% CI: 1.28, 9.88; *P* = .02). Time from diagnosis • Median time to VTE = 3.8 mo
Harza et al. [6] Romania^b^	2013	Prospective Observational	Yes	191	Mixed MN 56 (29%) FSGS 47 (25%) MCD 25 (13%) IgAN 34 (18%) MPGN 29 (15%)	Prevalence: VTE – 23 (12%) MN 11 (20%) FSGS 5 (11%) MCD 1 (4%) IgAN 2 (6%) MPGN 4 (14%)	Time from diagnosis • 65.2% of VTE occurred within 6 mo Histologic subtype • MN having highest incidence of 11.5% per 100 pt y
Zhang et al. [7] China^b^	2014	Prospective CTA	Yes	512	Mixed MN 183 (36%) FSGS (7%) MCD (2%) Podocytopathy (45%) Others	Prevalence VTE – 180 (35%) PE – 153 (85 with RVT) – 30% 84% of PE were asymptomatic	Age • >60 y, Histologic subtype • MN, were independent risk factors in multivariate analysis (*P* < .05)
Li et al. [8] China^c^	2016	Prospective Screening CTA	Yes	120 70 Incident 50 Relapse	FSGS	Prevalence VTE – 10% (n-=12) PE – 8 (3 asymptomatic) RVT – 4	
Maas et al. [9] Netherlands^d^	2017	Retrospective 10 Centers	Yes	125	MCD	Prevalence TEs – 11 (9%) VTE – 9 (7%) ATE – 2 (2%)	Time from diagnosis • 9 of 11 events occurred within 17 days of presentation with NS
Gyamlani et al. [10] US^b^	2017	Prospective Prevalent population	Yes	7037 US veterans	Mixed 26% with Glomerulonephritis 52% DN	VTE – 158 (2.2%) over 8 years Incidence 3.2 per 1000 patient years	Albumin • Serum albumin <2.5 g/dL had adjusted HR: 2.79 (95% CI: 1.45-5.37) of VTE compared with patients with serum albumin >4 g/dL
Fenton et al. [11] UK^d^	2018	Retrospective Single center	52 (available data) – Yes	78 61 Incident 17 Relapses	MCD	VTE – 9 (12%)	
Zou et al. [12] China^a^	2018	Retrospective	69% of cohort	766	MN	Incidence VTE – 6% at 6 mo, 7% at 1 year ATE – 4% and 6%, respectively	From multivariate analysis, risk factors for VTE serum albumin • Albumin <20 g/L compared with >32 g/L had an HR = 3.2; (95% CI: 0.8-12.5; *P* = .03) Time from diagnosis • 77.4% VTE occurred in 6 mo Risk factor for ATE Age >55 years compared <35 y HR 9.2 (95% CI: 2.2-39.5; *P* = .001)
Shinkawa et al. [13] Japan^b^	2021	Retrospective Admin database	Yes	6866 Hospitalized with NS	Mixed MN 11% MCD 18% FSGS 2%	Incidence VTE – 221 (3%)	From multivariate analysis significant risks Acute kidney injury • OR = 2.15 (1.34-3.46) Body mass index • ≥30 (vs <25) OR = 1.77 (95% CI: 1.12-2.80) Sepsis • OR = 2.49 (1.09-5.66)
Vestergaard et al. [14] Denmark^b^	2022	Retrospective Nationwide Registries	Yes	3967 1:10 matching	Mixed Only 50% with biopsy proven KD MN (455) 25% FSGS (163) 8.5% MCD (468) 25%	Incidence ATE – 10 y 14% VTE – 10 y 7.7% ATE – 1 y – 4.2% VTE – 1 y – 2.8%	Not reported

CTA, computed tomography angiography; FSGS, focal segmental glomerulosclerosis; HR, hazard ratio; IgAN, IgA nephropathy; MCD, minimal change disease; MN, membranous nephropathy; MPGN, membranoproliferative glomerulonephritis; NS, nephrotic syndrome; OR, odds ratio; PE, pulmonary embolism; PT, prothrombin time; RR, relative risk; RVT, renal vein thrombosis; VTE, venous thromboembolism.

a MN study.

b Mixed NS study.

c FSGS study.

d MCD study.

Risk of thromboembolism differs with the primary cause of NS, with the greatest risk seen in membranous nephropathy (MN); 36% of patients were identified with VTE on screening in studies using contrast enhanced computed tomography [2,7]. Barbour et al. [4] demonstrated a 2-fold increased risk of VTE in patients with MN compared with other causes of glomerulonephritis. It must be noted that patients with cancer were included in this study. However, after multivariable analysis, MN still had the highest risk with an adjusted hazard ratio of 10.8 when compared with IgA nephropathy. More recent studies of minimal change disease showed incidence rates of VTE between 9% and 12%, compared with 24% in previous studies [2,4,6,[16], [17], [18], [19], [20], [21], [22], [23]]. VTE was noted in 10% of patients with focal segmental glomerulonephritis (FSGS) [4,6,8].

Arterial thromboembolism (ATE) is less well reported. A study by Mahmoodi et al. [24] found absolute risk of ATE (1.48% per year) that was approximately 8 times higher in patients with NS than estimated age- and sex-weighted incidences in the general population of the Framingham study. This risk was particularly high within the first 6 months of NS diagnosis with an annual incidence of 5.52% (95% CI: 2.38-10.87). The high rates of ATE within the first 6 months of diagnosis may relate to transient risks, such as hyperlipidemia (92%), proteinuria (8.1±5.2 g/d), hypertension (61%), and steroid use [24]. The population in this study also had other significant risks, including 50% of patients with a smoking history, renal impairment (mean estimated glomerular filtration rate of 59 mL/min/1.73 m^2^) and 9% having already experienced a previous event [24]. Prior to the study by Mahmoodi et al. [24], limited small case series demonstrated conflicting results of whether the risks of ATE were increased [25,26]. A larger more recent study by Lee et al. [27] found that the observed cumulative incidences of cardiovascular events (myocardial infarction and ischemia, ischemic cerebrovascular events, and peripheral artery occlusion disease) were 4.4% and 5.4% for year 1 and 2, respectively, after MN diagnosis. They found early in the course of MN that the incidence of cardiovascular events was similar or exceeded that of those with end-stage renal disease [27]. The risk factors in this study were similar to the study by Mahmoodi et al. [27], with mean cholesterol 338 mg/dL, proteinuria 8.7 g/day, 33% with smoking history, 12% with previous ATE, and 76% taking steroids.

## 2 What is the Cause of VTE in NS?

**Figure 1:**
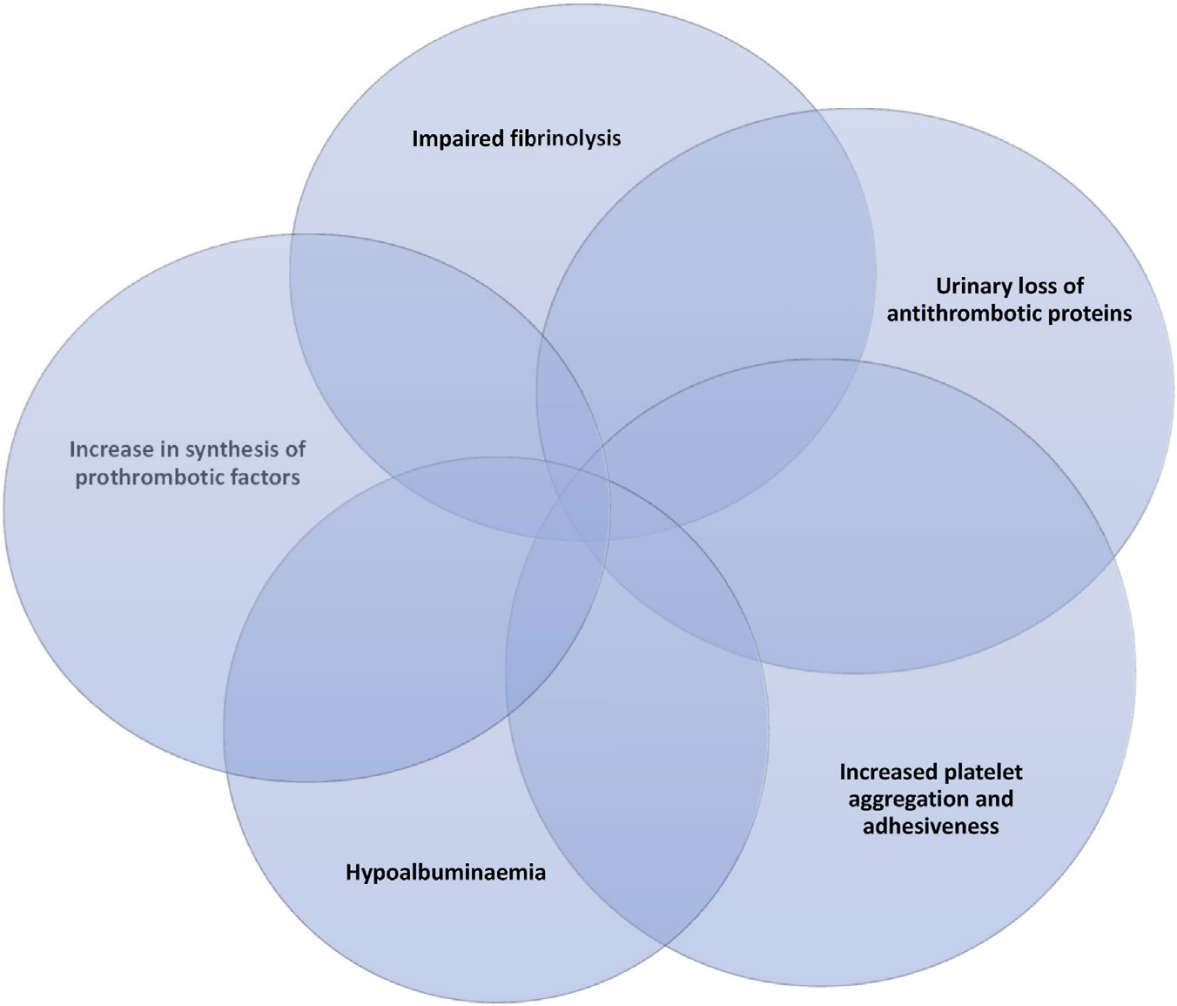
Factors leading to hypercoagulability in nephrotic syndrome.

The pathophysiology of hypercoagulopathy and risk of VTE in NS is not well understood (Figure 1). It is believed to be related to an increased synthesis of prothrombotic factors, eg, increased fibrinogen, factor VIII (FVIII) levels, these can act as acute phase proteins and are upregulated in inflammation. This is alongside urinary losses of negatively charged anticoagulant proteins such as antithrombin, protein C and S leading to reduction in their levels, and impaired fibrinolytic activity (decreased plasminogen levels, elevated plasminogen activator inhibitor-1 levels or albumin deficiency-related impairment of the interaction of plasminogen-fibrin). The loss of albumin and resultant hypoalbuminemia results in increased hepatic synthesis of fibrinogen and other procoagulant factors [28,29]. One study has used deficiency in coagulation inhibitors and anomalies of the fibrinolytic pathway as a biological marker for the risk of thrombosis [30].

In a study by Andrassy et al. [31], patients with plasma albumin levels <20 g/L showed significant reductions in plasma antithrombin levels, with a further study finding that a low serum albumin was correlated with low plasma antithrombin levels [32]. However, Li et al. [8] found that antithrombin deficiency, defined as antithrombin <25 mg/dL, was common (>80%) in patients with NS secondary to FSGS, but this did not correlate with VTE events.

The tendency for thrombus formation in the renal vein may in part be due to intravascular volume depletion leading to hemoconcentration in the postglomerular circulation [16] and to the loss of coagulation inhibitors (protein C and S and antithrombin) via urine, which may make the renal veins particularly hypercoagulable. Further factors to be considered include volume depletion related to diuretics, steroid therapy, venous stasis, immobilization, or immune complex activation of the clotting cascade [15].

Abnormalities are also found in platelet function with reported increased aggregation and adhesiveness in NS, which may play a role in promoting thrombotic complications [31]. Albumin loss results in increased free arachidonic acid and formation of thromboxane A2, which may be responsible for platelet hyperaggregability [33,34]. Andrassy et al. [31] found that platelet aggregability correlated with plasma cholesterol levels in patients with NS, and hyperlipidemia is well known to affect platelet function [33,35].

The detailed pathophysiology is beyond the scope of this article and has been reviewed previously [16,33,36]. However, determining the key drivers for thromboembolism is required to understand this complex disease pathophysiology, to pave the way for better prediction and diagnostic tools.

## 3 What are the Risk Factors for Thromboembolism?

To date, factors involved in the increased risk of thromboembolism in NS have not been fully elucidated. In addition to traditional risk factors for VTE [37], other proposed independent risks of VTE in NS are hypoalbuminemia, MN, high-urine protein excretion, and the 6-month period within NS diagnosis. The risk of ATE appears to be related to traditional risk factors, including hyperlipidemia. Studies detailing the risks are discussed.

### 3.1 VTE risk factors

#### 3.1.1 Histopathology

As described, the histologic subtype of NS influences the incidence of VTE, with membranous having the highest rates [4,6,7,24,38]. This risk remains despite adjustment for baseline factors, including proteinuria, serum albumin, and age. The reasons why this occurs are uncertain. However, recent analysis of 83 patients with MN suggested that presence of anti-PLA2R may be associated with a higher risk of thrombosis [39]. For patients with anti-PLA2R, 20.5% developed VTE compared with 9.1% who were seronegative for anti-PLA2R. Four patients developed VTE despite serum albumin levels >25 g/L, all of these were anti-PLA2R positive [39]. This needs further investigation in larger studies to determine if this finding is consistent, but it raises questions as to whether anti-PLA2R plays a role or is a biomarker in development of VTE.

#### 3.1.2 Age

Age is an important risk factor for developing VTE, and this has also been shown to be the case in NS. Adults have approximately a 7- to 8-fold increased incidence of thrombosis compared with children [36]. Zhang et al. [7] demonstrated that >60 years of age was an independent risk factor (*P* < .05) for VTE in patients with NS. Older age is a known risk factor for VTE, which may relate to enhanced coagulation activation and decreased fibrinolytic activity with older age, alongside reduced mobility and increased number of comorbid conditions [40].

#### 3.1.3 Urine protein excretion

High rates of urinary protein excretion are deemed to be associated with an increased incidence of thrombotic events in patients with NS. Kumar et al. [3] retrospectively studied 78 patients with primary MN and found that patients with VTE had more proteinuria, 10.7 g/dL/d, than patients without VTE, 7.1 g/dL/d, with an odds ratio for developing VTE of 1.3 (95% CI, 1.05-1.58; *P* = .01) for each increase of 1 g/d of proteinuria. Kato et al. [41] found patients with MN who had proteinuria, compared with patients without, had a 3.4-fold increased risk for developing VTE, and higher levels of proteinuria (2+ and 3+) were associated with higher likelihood of VTE compared with lower levels of proteinuria (1+). This association was unchanged when adjusted for other risk factors. Another study by Li et al. [2] showed that in 100 patients with MN, those with thrombus had higher proteinuria (7.98 g/d) compared with those without thrombus burden (6.39 g/d) and odds ratio was 1.18 (95% CI, 1.02-1.35; *P* = .02) for developing VTE.

#### 3.1.4 Serum albumin

Multiple studies identify serum albumin level as an independent risk factor for thrombotic events in NS. The severity of hypoalbuminemia is believed to be a surrogate marker for the degree of imbalance in prothrombotic and antithrombotic factors, although VTE can occur even when the serum albumin is only moderately reduced [15]. Lionaki et al. [5] studied 898 patients with biopsy proven MN and reported below an albumin threshold of 28 g/L, there was a 3.9-fold increased VTE risk, rising to 5.8-fold when serum albumin was below 22 g/L. Kumar et al. [3] retrospectively studied 78 patients with idiopathic MN. Patients with VTE had lower serum albumin concentrations (19 ± 5 g/L) than patients without VTE (24 ± 4 g/L). Bellomo and Atkins [22] found that VTE events occurred in 40% of patients with MN and NS with a serum albumin concentration of ≤25 g/L but only 2.7% in those with a serum albumin concentration of >25g/L. A study by Mahmoodi et al. [24] found an inverse association between albumin level and risk of VTE in almost 300 patients with various causes of NS, although this was not significant. A large prospective study in the United States found lower serum albumin was associated with an increased risk of VTE in patients with NS, increasing proportionally with reduced albumin levels [10].

It should be noted that studies used different assays for the measurement of albumin with some not detailing the assay [3,5,10,24], hence drawing a conclusion on specific albumin cut-off is difficult. Assays differ with higher albumin values reported with bromocresol green than with bromocresol purple and immunoassays [42]. The current Kidney Disease Improving Global Outcomes (KDIGO) guidelines suggest to use bromocresol purple and immunoassay [37].

#### 3.1.5 Time from diagnosis

In a retrospective cohort study of 298 patients with NS, VTE developed within the first 6 months in 9.85% of the patients, which compares to annual incidence rates of 1.02% over a 10-year follow-up period [24]. Kumar et al. [3] found that of the 20% of membranous patients who developed a VTE, 64% had a VTE at presentation and the remainder developed VTE within 6 months of diagnosis. While Harza et al. [6] found that the risk of VTE was highest in the first 6 months of presentation with a median follow-up of 24 months (mixed causes of NS).

Lionaki et al. [5] found in MN that the median time to VTE occurrence was 3.8 months. However, despite most patients developing VTE within 2 years, 26% of events occurred *after* this time. Barbour et al. [4] found that the median time to first VTE was 272 days, but this differed based on the primary diagnoses. VTE events in MN presented at a median 151 days which was 1094 days in FSGS.

### 3.2 ATE

There is less literature relating to ATE. Traditional risk factors, such as sex, age, hypertension, diabetes, smoking, prior ATE, and reduced eGFR, were significantly associated with ATE in a study of patients with NS undertaken by Mahmoodi et al. [24]. This study showed no association between ATE events and degree of proteinuria or serum albumin levels. In contrast, a larger study by Lee et al. [27] using 2 large cohorts of patients with MN, one as a discovery cohort and the other as an external validation cohort, found cardiovascular events within the first 2 years had a correlation with proteinuria and hypoalbuminemia. However, after 2 years, the events were not related to the nephrotic state.

Table includes a summary of the risk factors for VTE and ATE in the most recently published NS studies.

## 4 Who Should Receive Prophylactic Anticoagulation?

Despite thromboembolic events being a preventable cause of morbidity in patients with NS, prophylactic anticoagulation is not universally used. Prophylactic anticoagulation was not used in patients with MN in the *Glomerular Disease Collaborative Network* between 1969 and 2007 or the *Toronto GN Registry* between 1974 and 2005. In 2012, Kerlin et al. [36] suggested that there was insufficient evidence to initiate prophylactic anticoagulation in patients with NS. There are few studies that report on the outcomes for patients with NS who are treated with prophylactic anticoagulation.

Currently, decisions around prophylactic anticoagulation for patients with NS are made on a case-by-case basis with assessment of thrombotic and bleeding risk factors. Traditional risk factors such as obesity (body mass index of >35 kg/m^2^) and prolonged immobilization also need to be considered [37]. The uncertainty with use of anticoagulation was demonstrated in a European survey undertaken by the *European Rare Kidney Disease Registry* (ERKnet) in 2019 (personal correspondence with Professor Wetzels), where 27% of specialist units did not routinely use any prophylactic anticoagulation. Similar variation was seen in a recent United Kingdom survey where varying levels of albumin cut-off were used to decide upon prophylactic anticoagulation, with other risks factors, such as primary cause of NS and degree of proteinuria not always being included in the decision [43].

Following a systematic review of the literature, Lin et al. [38] proposed an algorithm for initiation of prophylactic anticoagulation in patients with NS. They consider the primary cause of NS, serum albumin, and bleeding risk when deciding upon whether to initiate therapy [38]. The recently updated KDIGO guidelines suggest that anticoagulation should be considered if serum albumin is <20 to 25 g/L with additional risks for thrombosis which include proteinuria >10 g/d, body mass index of >35 kg/m^2^, family history of thromboembolism with documented genetic predisposition or other traditional risk factors [37]. This decision would depend on the bleeding risk [37]. The algorithm proposed in the KDIGO guidelines has been based on patients with MN, which leaves clinicians faced with uncertainty of the usefulness of the algorithm for other causes of NS.

In 1994, Sarasin and Schifferli [44] developed a Markov-based–decision-analysis model considering embolic and bleeding risks for use of prophylactic anticoagulation. Despite numerous assumptions within the model, they concluded for patients with MN and NS that the benefits of prophylactic anticoagulation outweighed the risks [44]. Lee et al. [45] constructed a Markov decision model regarding the initiation of prophylactic anticoagulation in patients with MN. They used a cohort of 898 patients with MN to calculate VTE risk, with bleeding risk taken from published studies. Lee et al. [45] suggest that any patient deemed to be at high-bleeding risk is unlikely to benefit from prophylactic anticoagulation. The benefit-to-risk ratio of warfarin to prevent VTE events was based on serum albumin level and compared to the patient’s estimated bleeding risk. This has now been developed into a tool suggested by KDIGO (http://www.med.unc.edu/gntools/gntools-team.html), within this tool the ATRIA bleeding risk score is used to identify factors to consider in relation to bleeding risk [46]. In clinical practice, this tool has limitations in that it is only validated for MN, therefore, its value for other histologic subtypes is uncertain. It also asks the user what benefit-to-risk ratio would be considered acceptable, which can potentially lead to inconsistency, despite discussion with patients to arrive at a shared decision.

**Figure 2:**
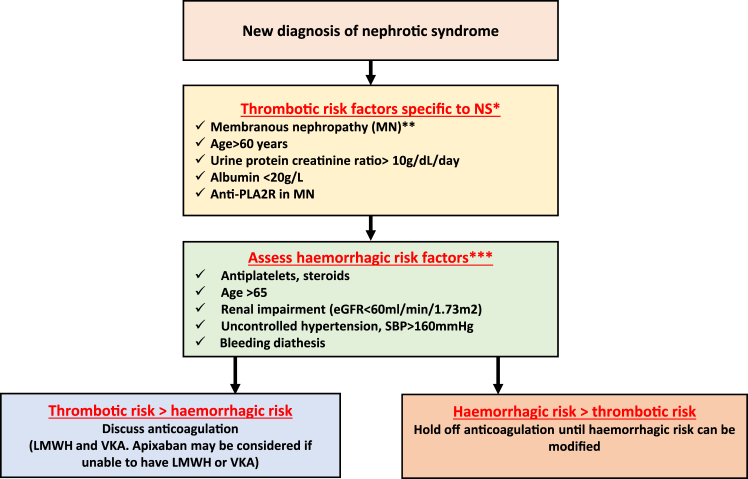
Decision making around the use of anticoagulation in Nephrotic syndrome. LMWH, low molecular weight heparin; SBP, systolic blood pressure; VKA, vitamin K antagonist.

Bleeding rates have been reported in patients with NS by a large Danish study [14]. This study found the adjusted HR for bleeding was 4.02 (95% CI, 3.40-4.75), using the general population as a comparator group controlling for confounders, such as age, sex, medicines, and comorbidities [14]. Gastrointestinal bleeding was the most reported bleeding event. The reasons for increased rates of bleeding are still poorly understood and further research is warranted to identify specific risk factors; however, the use of conventional bleeding risk scores could be used in clinical practice. The authors use the HASBLED (hypertension = 1, age >65 = 2, stroke history = 1, renal disease = 1, liver disease = 1, labile international normalized ratio [INR] = 1, ethanol = 1, drugs = 1) [47] score to assess risk factors for bleeding (Figure 2).

Kelddal et al. [48] reported on anticoagulant practices in patients with NS across 2 Danish renal departments. One unit initiated prophylactic anticoagulation based on serum albumin levels <20 g/dL. While in the second department, prophylactic anticoagulation was initiated irrelevant of the albumin levels. They found no episodes of thromboembolism in the 44 patients on prophylactic anticoagulant whereas 4 patients out of 35 who did not receive prophylactic anticoagulation had thromboembolic events [48]. Five patients experienced bleeding events in the anticoagulated group vs 2 patients not receiving anticoagulation. The 2 patients who experienced major bleeding events were receiving both anticoagulants and antiplatelets. Although there was a reduction in thromboembolic events the authors caution of the possibility of increased bleeding [48].

Medjeral-Thomas et al. [49] retrospectively reported on the use of an anticoagulation regimen in 143 patients with either primary MN, minimal change disease, or FSGS over a 5-year period. Patients were excluded if there was contraindication to anticoagulation. They used serum albumin as a threshold for initiation of prophylactic anticoagulation. Patients with serum albumin <20 g/L received prophylactic low molecular weight heparin (LMWH), which could be changed to low-dose warfarin for a target INR of 1.5 to 2.5. They found that patients who had been on prophylactic anticoagulation for over a week did not experience VTE [49].

A study by Rostoker et al. [30] selected 10 patients with NS deemed at thrombotic risk based on biological markers (severe hypoalbuminemia, deficiency in coagulation inhibitors, and anomalies of the fibrinolytic pathway). They received prophylactic anticoagulation with no thrombotic events or notable side effects. The group then included an additional 55 patients who were treated for up to 6 months with prophylactic anticoagulation, with no reported VTE events or side effects. The authors suggest that prophylactic doses of LMWH are an appropriate strategy for preventing VTE in patients with NS.

Despite the very limited number of studies, serum albumin, although not the only risk factor, appears to have been used as the main surrogate marker to determine the use of prophylactic anticoagulation in patients with NS. This was also seen in the ERKnet and UK surveys with 50% and 100%, respectively, of units responding that they would use anticoagulation with a serum albumin levels of <20 g/L [43]. Given the bleeding potential in patients with NS, its assessment should also be undertaken and used as part of the decision-making process when initiating anticoagulation. Bleeding risk assessment was considered in anticoagulant decision making of 89% respondents from the UK survey [43]. The authors propose the factors to consider when making decisions about prophylactic anticoagulation in Figure 2.

## 5 What Anticoagulant should be Used for Prophylaxis?

If the use of prophylactic anticoagulation were to be decided, the next clinical conundrum is the most appropriate anticoagulant regime.

Medjeral-Thomas et al. [35] used 20 mg of enoxaparin subcutaneously (s.c.) daily with consideration for use of warfarin if the albumin level remained <20 g/L for >3 months. The target INR for warfarin was lower than usual with a range of 1.5 to 2.5. The patients changed treatment according to the serum albumin levels during follow-up and once albumin level was ≥20 g/L, patients were placed on aspirin. No VTE events occurred in patients on prophylaxis for >1 week, with a median follow-up of 154 weeks. Two patients developed embolic events within 7 days of diagnosis, but it was not clear if the thrombus had been formed before or after prophylaxis was initiated. One patient with anemia receiving enoxaparin required blood transfusion where no cause could be identified. The authors suggest this regime is effective with few complications. However, it should be noted that in Europe unless Cockcroft-Gault Creatinine Clearance <30 mL/min then the prophylactic dose of 40-mg enoxaparin is administered s.c. daily. The licensed prophylactic dose should be considered when initiating therapy.

The dose of 40 mg s.c. daily administration was used by Rostoker et al. [30] as prophylaxis for VTE. Moreover, 50% of patients (*n* = 30) received at least 6 months of this regime. There was no VTE or side effects in this study, and the authors suggest that prophylactic LMWH should be the first line anticoagulant regime in NS [50].

The study by Kelddal et al. [21] used a variety of anticoagulant regimes based on clinicians’ preference, thus allowing them to opt for low or high doses of LMWH or warfarin with/without LMWH bridging. Moreover, 36% of patients received prophylactic LMWH (*n* = 15) which included enoxaparin ≤40 mg of daily administration s.c. or dalteparin ≤5000 units daily administration s.c., with similar numbers initiated on warfarin with bridging LMWH. None of the patients taking prophylactic anticoagulation developed VTE over the median follow-up of 92 weeks. Although Kelddal et al. [21] had a group of patients not receiving anticoagulation, this group cannot be used for a comparator of outcomes because it included patients with albumin >2.0 g/dL from one hospital site. The differing anticoagulant regimes in this study make it difficult to determine the most appropriate regime.

Prophylactic dose LMWH is the most reported anticoagulant regime, with warfarin being used if NS was prolonged. There are several limitations to these studies in that they are small and mainly retrospective. Concerns have been raised about the potential resistance to LMWH due to urinary antithrombin losses although this has not been supported by the published studies. Another difficulty with long-term use of LMWH relates to the burden of regular injections for the patient. Owing to the high-protein binding of warfarin, dosing regimens for initiation may need to be reduced (eg, loading with 2-3 mg rather than 10 mg).

The use of direct oral anticoagulants (DOACs) for prophylaxis of VTE in NS is gaining more attention. A single report of 2 cases in which patients received 5 mg of apixaban twice daily until remission was achieved, was short lived due to rapid remission of NS (8-12 weeks), making this difficult to fully assess safety and efficacy [51]. Kelddal et al. [52] reported on 19 patients receiving either 5 mg of apixaban twice daily (unless 2 factors for dose reduction) or 20 mg of rivaroxaban daily, for primary prophylaxis of TE in those with serum albumin <25 g/L for MN or <20 g/L for other NS subtypes [52]. With a median treatment duration of 72 days, no TE events and 5 minor bleeding episodes were observed and the bleeding risk increased with treatment duration [52]. Van Meerhaeghe et al. [53] report 24 patients who received apixaban for primary prophylaxis in NS, with nearly 50% of patients having MN, using the same dosing of apixaban as Kelddal et al. [52]. Patients consumed apixaban for a mean period of 129 days with one patient developing pulmonary embolism during follow-up while on apixaban [53]. There were no major or minor bleeding episodes [53]. A further study reviewed patients receiving warfarin (*n* = 19) or apixaban/rivaroxaban for NS (*n* = 25) and compared bleeding and thrombotic events between the 2 [54]. One patient receiving apixaban developed deep vein thrombosis after 146 days of treatment. The warfarin group had higher numbers of major bleeds, with those with major bleeding having a time in therapeutic range between 28% and 47%. Owing to the small numbers in this study, the results were not significant, but the authors conclude that DOACs could be an option in NS. It should be noted that some patients in this study had other indications for anticoagulation, and patients were anticoagulated for diabetic nephropathy [54]. Therapeutic dosing is used in these studies, but it is uncertain if lower doses may be sufficient. Concerns relate to DOACs due to high-protein binding, with apixaban and rivaroxaban being around 90% protein bound. It is currently unclear whether there may be overexposure to a DOAC due to increased free drug levels or underexposure due to urinary losses of drug. Currently, a study is underway to determine apixaban pharmacokinetics in patients with NS receiving multiple doses compared with healthy volunteers (NCT04278729).

The ERKNet survey found for patients with serum albumin levels of 17 g/L, 7 of 11 specialist centers would use prophylactic warfarin, prophylactic LMWH, aspirin, or a combination of aspirin with prophylactic LMWH. Similar variability is presented in the United Kingdom survey, with the majority of centers using LMWH (therapeutic or prophylactic) or warfarin with albumin <20 g/L with some use of DOACs in this setting [43]. This highlights how the lack of evidence to support management has led to differing practice. The authors propose an algorithm for the use of anticoagulation in NS (Figure 2). Figure 2 suggests LMWH or warfarin as preferred agents based on limited available evidence and current practice. However, there may be consideration of apixaban as an alternative for those unable or unwilling to have LMWH or warfarin, until further information is available to support more widespread use of DOACs in this setting.

## 6 What about Antiplatelets?

Medjeral-Thomas et al. [35] used aspirin as VTE prophylaxis in patients with NS and an albumin level of 20 to 30 g/L. They based this decision on their assumption that platelet abnormalities in NS underpin their pivotal role in thrombus formation. They found that none of the patients experienced a VTE, but one patient receiving aspirin had a major gastrointestinal bleed whereas another patient had anemia of unknown cause requiring a blood transfusion. In contrast, the study by Lionaki et al. [5] on an MN population had 43% of patients who developed VTE while receiving aspirin. The use of aspirin for prevention of VTE is suggested in the current KDIGO guidelines for patients who are at high risk of bleeding as an alternative to anticoagulation [37]. Whether aspirin should be used in this context still remains unclear.

Based on the high rates of early cardiovascular events reported by Lee at al. [27], Hofstra and Wetzels [50] proposed an algorithm for the use of aspirin in primary prophylaxis of ATE in patients with MN, which is incorporated in the KDIGO glomerular diseases guideline. The KDIGO guidelines make recommendations about the use of aspirin in those deemed at a high risk of cardiovascular events, defined by the Framingham risk score [55]. The Framingham risk score is based on traditional factors for developing cardiovascular events such as diabetes, age, and high cholesterol, and these factors were also shown by Lee et al. [24] and Mahmoodi et al. [27] to be significant in the risk of ATE in patients with NS.

## 7 What do we Need to Improve Management of Patients with NS in the Future?

Multiple studies described in the present article illustrate varied incidence of thromboembolism events. So far, studies are yet to systematically evaluate the preventative strategies and their risks and benefits, which, therefore, beg the question what the optimal regime would be in clinical practice. We believe that until further evidence is accumulated, variation in practice is likely to remain with differences in patient outcomes.

KDIGO 2021 guidelines outline several aspects for evaluation in this area of thromboembolism in NS [37]. First, robust estimates of disease-specific absolute risk of thromboembolism (and association with clinico-demographic variables) are urgently needed. These can be possible by using multicenter and multinational observational registries with linkage to primary care and hospital statistics. Multicenter, subgroup prospective studies with deep phenotyping and genetic predisposition would guide further risk stratification.

From a United Kingdom perspective, we propose this could be undertaken by utilizing 3 to 5 years of prospective observation from the United Kingdom Kidney Association Rare Disease Registry alongside integration with Primary Care Information and Hospital Episode Statistic data. Similar registries such as ERKNET in Europe could be vital in such studies. Use of different anticoagulant prescribing practice would have to be accepted to provide a current perspective on VTE rates, the effect of which may be teased out using propensity matching studies.

Alongside the risk evaluation, examining the pathobiology behind the causation of TE will be vital to understanding varying risks posed by different glomerular diseases. With immune basis for MN becoming explicit since the discovery of PLA2R mechanism one wonders if there is an immunologic basis for higher risk of thromboembolism in MN. This may provide further clues to directed management of thromboembolism risk.

Equally important is the need to examine the role of DOACs in prevention of thromboembolism in NS, including pharmacokinetic studies, given their ease of use and safety in non-nephrotic thromboembolism.

## 8 Conclusion

The risk of VTE and ATE is significantly elevated in patients with NS, although the reported incidence varies widely between studies. Several risk factors are proposed, although these do not fully explain all thromboembolism events. Despite the existence of guidelines, uncertainty still exists as to who should receive prophylactic antiplatelets or anticoagulants, which agent to use, and when. Large registry and multicenter studies are needed to examine real-world and prospective data. This will help optimize future practice and improve patient outcomes in a key and potentially life-threatening complication with NS.
